# Scientific obituary: Peer Bork

**DOI:** 10.1038/s44320-026-00204-x

**Published:** 2026-03-24

**Authors:** Jan O Korbel

**Affiliations:** Interim Head of EMBL Heidelberg, European Molecular Biology Laboratory, Heidelberg, Germany

## How I met Peer

Writing these words about Peer Bork in the past tense still feels unnatural. His recent passing at the age of 62 has left a profound void in science and in the lives of those who had the privilege of working alongside him. For many, Peer was a visionary scientist whose work reshaped several fields in biology. For me, he was all of that, but also something deeply personal: a mentor and manager, a collaborator, a scientific compass, and also, a friend. His influence shaped not only entire research fields but also the intellectual journeys and careers of generations of scientists, including my own.

I had the privilege of collaborating with Peer for almost 26 years. I still remember clearly the first time I heard him present on his science at a conference in Berlin in the autumn of 2000. At the time, I was a student at the Technical University of Berlin, while Peer was already an established and widely recognised scientist. I sensed immediately his extraordinary inspiration, his clarity of thought, and had the unmistakable feeling that he was advancing science in a highly original way. Not long afterwards, Peer became my doctoral advisor. Very few people shape one’s scientific life as profoundly as Peer shaped mine.

## His scientific career in brief

Peer’s scientific career was remarkable in several ways: in its breadth, its depth, and in his foresight regarding future developments in biology. Born in 1963 in the former East Germany, he began his scientific career working with Jens Reich in Berlin at the Central Institute for Molecular Biology of the Academy of Sciences of the German Democratic Republic, where he completed his PhD research in biochemistry.

Following Germany’s reunification in 1990, Peer moved quickly. In 1991, he joined EMBL Heidelberg with an EMBO fellowship, working with Chris Sander. In doing so, he entered a research environment that he referred to as uniquely enabling, providing fertile ground for his talent and vision to pursue a seamless interdisciplinary integration of biology and computation.

Over more than three decades at EMBL, Peer produced groundbreaking science and contributed substantially to shaping the institution itself. He served for many years as Head of the Structural and Computational Biology Unit, and later became the Director of EMBL Heidelberg and thereafter EMBL’s Interim Director General.

## Scientific fields he shaped

Scientifically, Peer was one of the pioneers who helped establish computational biology as a major discipline in the life sciences. His contributions spanned an extraordinary range of topics, including and not limited to genome analysis, protein function and structure prediction, protein interaction networks, drug–target relationships, microbiome research, evolutionary biology, and planetary biology. Among many seminal contributions, Peer played a key role in developing computational approaches for predicting protein function and interaction networks, as well as for the analysis of metagenomes (the complete collection of genetic material from all organisms in a given environmental sample).

Peer’s scientific curiosity rarely stopped at established boundaries of fields. Long before research on microbiomes (the combined genetic material and ecological characteristics of microorganisms) became mainstream, he recognised the immense biological and medical importance of microbial communities. He helped demonstrate that microbiomes are not merely collections of microorganisms but dynamic ecosystems that influence environmental processes and human health. His work identifying gut microbial community structures, so-called enterotypes, changed how scientists conceptualise microbial variation among individuals. Also, his contributions to disease research using the analysis of microbiomes were remarkable. By identifying microbial markers associated with conditions such as colon or pancreatic cancer and obesity, his research has helped open new avenues for diagnostic and therapeutic exploration.

One of Peer’s most inspiring initiatives was the Traversing European Coastlines (TREC) project, an initiative that drew inspiration from the Tara Oceans project, in which Peer had been involved from the very beginning. TREC is bringing together scientists from diverse disciplines to systematically study Europe’s coastal ecosystems while also engaging the public in scientific discovery. Peer designed, kick-started, and led TREC as its scientific director until his recent passing. Peer often drew parallels between ecological and microbial communities, seeing both as interconnected systems whose complexity demands collaborative exploration to gain a better understanding of our planet. Today, the analysis of thousands of samples generated through the TREC project continues as part of Peer’s enduring legacy.

## The personal side of an exceptional scientist

Peer’s approach to science was deeply intertwined with his personality. He embodied the philosophy of “work hard and play hard” in the truest sense. He was both a competitive scientist and an enthusiastic gamer. Free time was rarely spent idly, unless there was an opportunity for Peer to travel somewhere new in the world, which he eagerly pursued with his family.

At EMBL, his research group became notorious for dedicated computer gaming sessions right after lunch, with Peer often right in the middle of the action. I experienced this myself while working in his group two decades ago. I remember the joy in his eyes when he won a computer game, often succeeding in situations that at first seemed hopeless for him, and later taking pleasure in talking through what allowed him to secure his win with the lab.

These characteristics of Peer’s personality reflected his scientific spirit. His constant desire to explore novel data types, push established field boundaries through landmark publications, his careful effort to refine scientific narratives until they “got the point right”, and to ultimately go with his research where no one had gone before.

## Awards and recognition, and Peer as a mentor

Peer’s achievements were recognised globally. He received numerous prestigious awards, including the Novozymes Prize, the ISCB Accomplishments by a Senior Scientist Award, and the Royal Society and Académie des Sciences Microsoft Award. He was elected to Germany’s National Academy of Sciences, to EMBO, and to the Academia Europaea, and he held honorary doctorates and professorships across Europe and Asia. Peer served on the Editorial Board of numerous scientific journals, including the board of reviewing editors of *Science*, and the Editorial Board of *Cell*. For *Molecular Systems Biology*, he was one of the Founding editors. He secured two ERC Advanced Grants, and co-founded several successful biotech companies. In spite of this, however, it became clear from my interactions with Peer that what he was most proud of was his science and, above all, of the people around him, including the young scientists he advised and his collaborators.

Peer had a remarkable ability to identify potential in young scientists, often before they recognised it themselves. He encouraged intellectual risk-taking and fostered an environment in which curiosity was valued over convention. He reminded students, postdocs and colleagues to pursue the most meaningful questions rather than the safest or most fashionable ones.

For Peer, mentoring was not simply about completing projects but about enabling young researchers to develop independent scientific identities. Alumni from his group feel this connection for many years. Peer took great pride in staying in touch with them, often speaking about the successes of his alumni and inviting them to join him for scientific meetings and conferences long after they had established independent careers, as well as keeping them informed about developments at EMBL and in his research. The success of this philosophy is evident in the many scientists who trained under him and later assumed leadership roles across academia and industry. Peer’s mentorship was recognised with the *Nature* Award for Creative Mentoring in 2008.

I feel deeply privileged to have experienced his mentorship firsthand. It was my first meeting with Peer in Berlin that led me to the EMBL. Peer shaped how I think about scientific problems, how I evaluate evidence, and how I aim to balance ambition with intellectual integrity.

## Peer’s legacy

Beyond his own laboratory and the conference halls, Peer had a remarkable ability to build collaborative communities. For him, EMBL was never merely an institution. It was a living network of people united by curiosity and shared purposes. Peer’s legacy is visible everywhere: in the tools and databases we use every day, in the conceptual frameworks guiding systems biology and microbiome research, and in the collaborative and interdisciplinary ethos at EMBL.

Peer’s sudden passing has been deeply felt across the global scientific community. For those of us who worked closely with him, the loss is intensely personal. I personally already feel how much I miss him. In many conversations over the past weeks, with colleagues and especially with the young scientists he mentored, it has become clear how far his influence reached and continues to reach. His spirit will live on in our science, in the questions that continue to be asked, and in the people he inspired.

As we reflect on Peer’s life, it is impossible not to feel both deep sadness and gratitude. Sadness for the loss of a friend and colleague, and for the discoveries and conversations that now will not occur. But also gratitude for the extraordinary human and scientific legacy he leaves behind. Peer often emphasised that science is a collective and generational endeavour. In that sense, Peer’s work continues, in the people he inspired, and through the collaborations he nurtured.

His absence will be felt for many years to come. But Peer’s influence—on science, on EMBL, and on the many people whose careers he shaped and whose curiosity he instilled —will endure for far longer.

**Figure:**
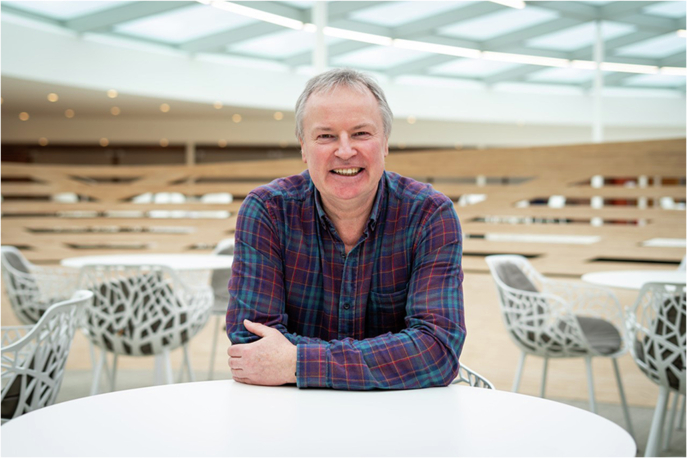
Photograph by Jeff Dowling/EMBL-EBI. Licensed under CC BY-SA 3.0 IGO

